# Generation of PHB from Spent Sulfite Liquor Using Halophilic Microorganisms

**DOI:** 10.3390/microorganisms3020268

**Published:** 2015-06-08

**Authors:** Michaela Weissgram, Janina Gstöttner, Bettina Lorantfy, Raimund Tenhaken, Christoph Herwig, Hedda K. Weber

**Affiliations:** 1 Kompetenzzentrum Holz GmbH, Altenbergerstraße 69, Linz 4040, Austria; E-Mails: michaela.weissgram@tuwien.ac.at (M.W.); janina.gstoettner@gmail.com (J.G.); h.weber@kplus-wood.ac.at (H.K.W.); 2 Institute of Chemical Engineering, Research Area Biochemical Engineering, Vienna University of Technology, Gumpendorferstraße 1a, Vienna 1060, Austria; E-Mail: lorantfy@chalmers.se; 3 Department of Cell Biology, University of Salzburg, Hellbrunnerstr 34, Salzburg 5020, Austria; E-Mail: raimund.tenhaken@sbg.ac.at

**Keywords:** polyhydroxybutyrate, halophilic microorganisms, halophilic archaea, spent sulfite liquor

## Abstract

Halophilic microorganisms thrive at elevated concentrations of sodium chloride up to saturation and are capable of growing on a wide variety of carbon sources like various organic acids, hexose and also pentose sugars. Hence, the biotechnological application of these microorganisms can cover many aspects, such as the treatment of hypersaline waste streams of different origin. Due to the fact that the high osmotic pressure of hypersaline environments reduces the risk of contamination, the capacity for cost-effective non-sterile cultivation can make extreme halophilic microorganisms potentially valuable organisms for biotechnological applications. In this contribution, the stepwise use of screening approaches, employing design of experiment (DoE) on model media and subsequently using industrial waste as substrate have been implemented to investigate the applicability of halophiles to generate PHB from the industrial waste stream spent sulfite liquor (SSL). The production of PHB on model media as well as dilutions of industrial substrate in a complex medium has been screened for by fluorescence microscopy using Nile Blue staining. Screening was used to investigate the ability of halophilic microorganisms to withstand the inhibiting substances of the waste stream without negatively affecting PHB production. It could be shown that neither single inhibiting substances nor a mixture thereof inhibited growth in the investigated range, hence, leaving the question on the inhibiting mechanisms open. However, it could be demonstrated that some haloarchaea and halophilic bacteria are able to produce PHB when cultivated on 3.3% w/w dry matter spent sulfite liquor, whereas *H. halophila* was even able to thrive on 6.6% w/w dry matter spent sulfite liquor and still produce PHB.

## 1. Introduction

In this project, the feasibility of a completely novel concept was investigated for a waste to value approach using spent sulfite liquor (SSL) as feedstock. The following novelties are claimed:

- Utilization of SSL by halophilic and extremely halophilic microorganisms.
- Generation of PHB using SSL as feedstock.
- Investigating the effect of common inhibitors in wood hydrolysate on Halophiles.

### 1.1. Characterization and State of the Art Utilization of SSL from Chemical Pulping

During the sulfite process, wood chips are delignified by cooking them with acid bisulfite. The generated waste water is called spent sulfite liquor (SSL) and contains dissolved solids such as lignosulfonates and hemicellulose hydrolysis products, which are comprised of about 30–35 g·L^−1^ hexoses and pentoses.

The composition of the sugar fraction in SSL depends on the type of wood used for pulping. Coniferous “soft” wood yields a high proportion of hexose sugars (predominantly mannose and glucose), whereas deciduous “hard” woods yield a high proportion of the pentose sugar xylose (Table 1).

As shown, waste streams from the pulping industry contain a high organic load, comprised of mono- and oligosaccharides and organic acids. Thus, they can act as a potential feedstock for bioprocesses. The SSL used throughout this study was derived from spruce. The only deviations from literature values were a lower galactose and higher mannose value.

### 1.2. Biorefinery on SSL

The high organic load in waste streams from the pulping industry can be a crucial parameter for industrial wastewater management. Analogously to the widely used process integration principles in the chemical industry, a biorefinery “waste to value” concept utilizing waste carbohydrates for the production of valuable chemicals could lead to process intensification, by coupling process effluent streams.

**Table 1:** Exemplary list of components in spent sulfite liquor from a softwood and a hardwood species, given in % (w/w) of dry matter (DM) [1,2,3].

	Sulfite Process	
pH	1–4	
temperature	80–95 °C	
	spruce % w/w DM	birch % w/w DM
monosaccharides		
arabinose	1	0
galactose	5	1
glucose	4	1
mannose	12	6
xylose	6	21
organic acids		
acetic acid	4	
aldonic acids	5	
glucuronic acid		2
others		
lignosulfonate	55	

Concepts using yeasts for the production of ethanol from spent sulfite liquor (SSL) have been used since the early 20th century. In 1909, the first sulfite ethanol plant opened in Skutskär, Sweden. Although sulfite ethanol mills have been built all around the world, many had to be closed down due to low fossil energy prices, high costs for substrate pretreatment, and the inability of non-genetically modified yeasts to ferment the highly abundant C5 sugar xylose.

**Figure 1:**
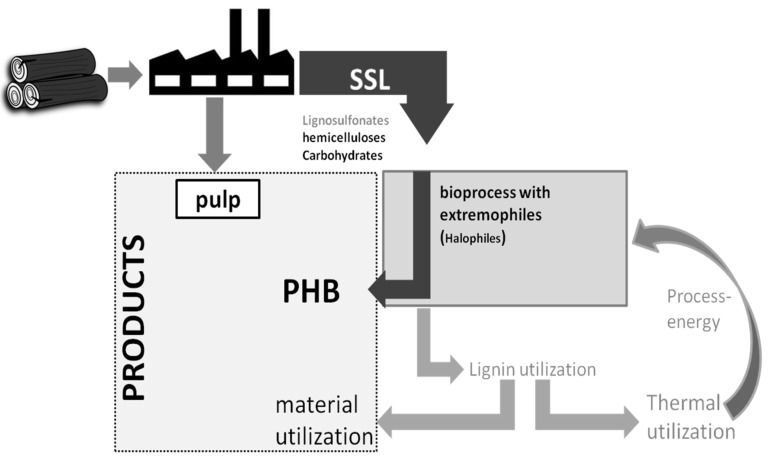
Proposed bioprocess using a waste to value approach using halophilic microorganisms.

Therefore, the utilization of lignocellulosic waste for the production of various chemicals has been investigated. Among them are, besides the already mentioned ethanol, butanol [4,5] and chemical building blocks, like lactic acid [6,7] and succinic acid [8]. We propose that other products with high market value could also be potential target candidates for the utilization of spent sulfite liquor (Figure 1).

**Figure 2:**
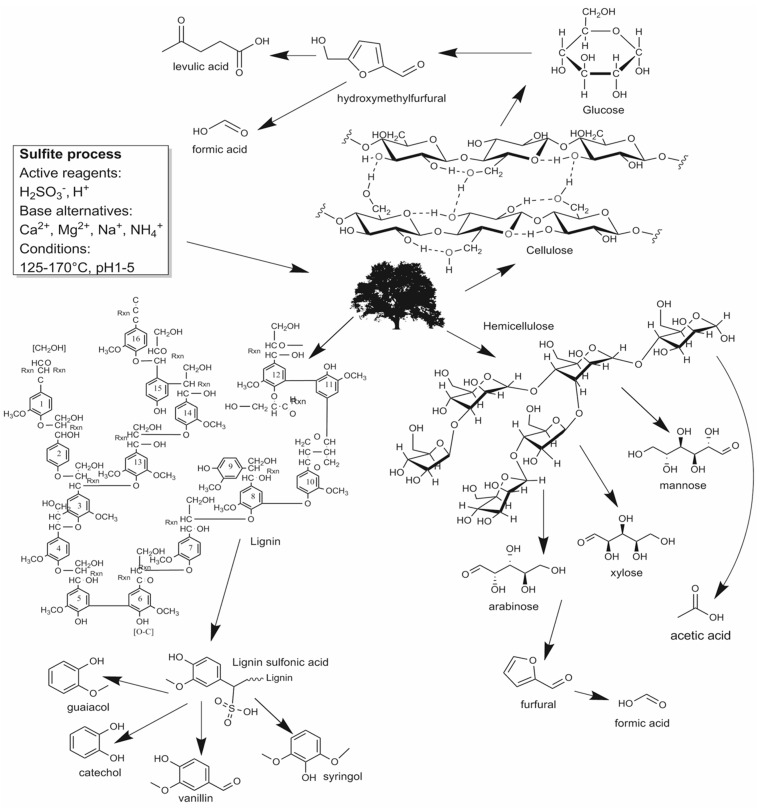
Typical scheme of acid hydrolysis of wood material showing the compounds that are potentially suitable as feedstock for halophiles [19,20,21].

Pulping wastewaters often contain high amounts of salts and metallic cations. Therefore, particularly, products generated by halophilic microorganisms are promising candidates for waste to value applications for spent sulfite liquor. Example products are betaine, ectoine, bioplastics like polyhydroxybutyrate, polyhydroxyvalerate, mixtures thereof, or other polyhydroxyalkanoates [9,10,11]. Halophilic microorganisms thrive at elevated concentrations of sodium chloride and are capable of growing on a wide variety of carbon sources. Among them are various organic acids like acetate [12,13], lactate [14] formate [15,16], and hexose [17] as well as pentose sugars, and even some phenols [18]. Many of these substances are present in typical SSL (Figure 2).

The typical composition of various spent sulfite liquors has been widely discussed in the literature (see Table 1) [1,2,3].

### 1.3. Common Substances in Lignocellulose Hydrolysate, Known to Inhibit Microbial Growth

Pulping wastewaters contain several wood degradation products that have been reported to inhibit microbial growth. Their effect on mesophilic microorganism has been widely discussed before [5,19,22,23,24,25]. A general description of inhibitors found in lignocellulose waste streams separated the inhibitors into four groups: organic acids, phenols, furan derivatives and metallic cations [25]. The furan derivatives furfural and hydroxymethylfurfural (HMF) are degradation products of pentoses and hexoses that can be further degraded to formic acid and levulic acid. Phenols are commonly found in wood and generated during lignin degradation. Large amounts of metallic cations are introduced during the wood hydrolysis, and again when adjusting the pH for fermentation. These cations are often calcium or magnesium, depending on the used base.

### 1.4. Halophilic Microorganisms in Biotechnology

Halotolerant or halophilic microorganisms, which are able to live in saline environments, offer several applications in various fields of biotechnology. They often produce compatible solutes, which are used as stabilizers of biomolecules and whole cells, play a role as salt antagonists, or can be used as stress-protective agents [26]. Examples for such compatible solutes are betaines, ectoines, or carotenoids [27]. Production of high value carotenoids, especially bacterioruberin, has been investigated [28,29]. Biopolymers, such as biosurfactants and exopolysaccharides (EPS), are of interest for microbially enhanced oil recovery. The degradation or transformation of a range of organic pollutants is another field of applications of this group of extremophiles [30]. Recent research on halophiles investigates their production of compostable bioplastics, mainly poly (3-hydroxybutyrate) (PHB) and other poly-hydroxy-alkanoates [31,32].

However, currently established applications of halophiles are limited primarily to the production of compatible solutes in the cosmetic industry, β-carotene and hydrolases in the nutritional and food industries [33].

The ecology and physiology of halophiles was mainly studied at the microbiological level, however, quantitative bioprocess development with extreme halophiles was also established recently, giving a methodological basis for quantitative bioprocess analysis of extreme halophilic *Archaea* [34].

The production of PHA by halophiles has been investigated before. The degradation or transformation of a range of organic pollutants is a field of applications of halophiles [30] and a wide range of biorefinery concepts for SSL are available. However, the linking concept to use halophiles for utilization of spent sulfite liquor (SSL) to propose a waste to value bioprocess is, to our knowledge, completely novel. The advantages of this application can be listed as working with a non-pathogenic, non-modified microorganism capable of utilizing a wide variety of organic compounds available in wood hydrolysate, that can be run under non-sterile conditions and yielding a high value product, which can be easily extracted during downstream processing.

The steps performed during this feasibility study included (1) the selection of suitable microorganisms; (2) the characterization of their carbon utilization spectra; (3) their resistance to dilutions of SSL as well as to (4) single and mixed substances commonly identified as inhibitors in fermentations on wood hydrolysate; and (5) a (purely qualitative) comparison of their production of PHB under ideal conditions and when cultivated on diluted SSL or glucose.

## 2. Experimental Section

### 2.1. Selection of Strains

Based on several factors a literature survey has been performed to screen candidates for utilization of SSL. The selected microorganisms are known to produce PHB and were reported to utilize preferably all the available sugars in SSL, or at least mannose, glucose and xylose, which comprise the major sugar fraction.

### 2.2. Media

All microorganisms were ordered from Deutsche Stammsammlung für Mikroorganismen und Zellkulturen (DSMZ, Braunschweig, Germany) and originally cultivated as indicated by DSMZ. For *Halomonas boliviensis* a second complex medium HM described by Quillaguaman 2004 [35] was used. *Haloferax mediterranei* was cultivated at pH 7.0 on medium containing NaCl 156 g·L^−1^, MgCl_2_ × 6 H_2_O 13 g·L^−1^, MgSO_4_ × 7H_2_O 20 g·L^−1^, CaCl_2_ × 2 H_2_O 0.67 g·L^−1^, KCl 4 g·L^−1^, NaHCO_3_ 0.2 g·L^−1^, NaBr 0.5 g·L^−1^, yeast extract 5 g·L^−1^, and glucose 1 g·L^−1^ [34].

### 2.3. Cultivation

All cultivations were performed in the respective medium in 100 mL shake flasks containing 20 mL of medium in a shaking incubator (Edmund Bühler GmbH, Hechingen, Germany) to ensure high oxygen transfer into the liquid phase. Cultivations were normally terminated after 44 h as literature suggested cultivation periods between 30 and 40 h for optimal PHB accumulation [36,37,38]. *Hfx. mediterranei* was cultivated for longer periods as it only enters the log phase after about 30 h.

### 2.4. Growth Measurement

The growth of the microorganisms was measured using OD measurements in microplates (100 μL volume/well) at 600 nm with a microplate spectrophotometer (Multiskan™ GO Microplate Spectrophotometer, Thermo scientific, Waltham, MA, USA), as this wavelength was confirmed to show lowest absorbance of diluted samples of pure SSL.

### 2.5. HPLC Analysis

For the quantification of sugars and metabolites, HPLC analysis with refractive index (RI) detection was performed (PerkinElmer Series 200 HPLC System, PerkinElmer Life and Analytical Sciences, Waltham, MA, USA), using a Supelcogel C610H column by Sigma Aldrich at 30 °C with a flow of 0.5 mL·min^−1^ and a mobile phase comprised of 0.1% H_3_PO_4_ in dH_2_O. Samples were centrifuged at 13,000 rpm (Hereus Biofuge fresco, Thermo Scientific, Waltham, MA, USA). The clear supernatant was then either frozen at −20 °C or diluted with dH_2_O and directly used for HPLC analysis.

### 2.6. Nile Blue Staining

Florescence microscopy of intracellular PHB was performed using the Nile Blue staining method described by Tekin *et al.*, 2012 [39]. Pictures were taken with a confocal microscope (Leica TCS5, Leica Microsystems, Wetzlar, Germany), using excitation at 488 nm (argon laser) and an emission window from 570 to 662 nm.

### 2.7. SSL

The used SSL came from a sulfite pulp mill using spruce as a feedstock. It had a dry content of 33.3% (w/w) dry matter, a pH around 4 and a total sugar content between 55 and 60 g·kg^−1^ with a ratio of 3.6:1.2:1 of mannose, xylose and glucose, respectively, with negligible amounts of arabinose, cellobiose, galactose, and rhamnose. All strains were tested for their resistance to dilutions of SSL. The respective media were supplemented with 3.33%, 6.66% and 16.65% (w/w) dry matter of SSL. The growth of cultures was monitored using OD, microscopy and HPLC analysis. All growing cultures were stained with Nile Blue to investigate the production of PHB.

### 2.8. Inhibiting Substances in SSL

Several inhibiting substances were tested for their effect on *H. halophila*. The inhibitors methanol, ethanol, formic acid, acetic acid, furfural and HMF were tested in concentration considerably higher than in the investigated dilutions of SSL. Vanillin was tested at a concentration of 10 mg·L^−1^as model for phenolic inhibitors and lignosulfonic acid was tested at 5 g·L^−1^, 10 g·L^−1^ and 25 g·L^−1^.

## 3. Results and Discussion

### 3.1. Strain Selection

Five strains have been selected according to their reported substrate utilization spectrum and their production of PHB (Table 2). These strains were further used for the screening.

### 3.2. Utilization of Various Carbon Sources

#### 3.2.1. Utilization of Single Sugars

Before carrying out fermentations on real substrate, the ability to utilize the main sugar in SSL reported in literature had to be confirmed experimentally for the used strains. The most abundant sugars in SSL are mannose, glucose and xylose, however, it also contains small amounts of arabinose, galactose and rhamnose and the disaccharide cellobiose.

All microorganisms were tested for utilization of all seven sugars. The initial sugar concentration was 5 g·L^−1^ of the respective sugar. In each sample, the investigated sugar was the only sugar supplemented to the medium, replacing the recommended medium sugar. The cultivations were run for 44 h. Subsequently, the bacterial growth on these sugars, as well as the uptake of the respective sugar was monitored by OD_600_ and HPLC measurement.

**Table 2:** Selected halophilic microorganisms and key parameters including their ability for utilization of arabinose (Ar), cellobiose (Cb), galactose (Ga), glucose (Gl), mannose (Ma), rhamnose (Rh) and xylose (Xy), as described in literature, with y indicating the utilized carbon sources of the strains

Name	Growth Conditions	Ar	Cb	Ga	Gl	Ma	Rh	Xy	Other	Product Yields
*Halomonas boliviensis* DSM 15516^T^	NaCl 0%–25% w/v, pH 6–11, 0–45 °C	y			y			y	oligo-sacharides Na- acetate, butyric acid	PHB (50%–90% w/w), ectoine (9.2 g·L^−1^) [35]
*Halomonas elongata* DSM 2581^T^	NaCl 0%–20% w/v, pH 5–10; 4–45 °C	y	y	y	y	y	y	y		PHB 55% (w/v); ectoine [35,40]
*Halomonas eurihalina* DSM-5720^T^	NaCl 0.5%–25% w/v pH 5–10, 4–45 °C	y	y	y	y	y	y	y		PHB [40]
*Halomonas halophila* DSM 4770	NaCl 2%–25% w/v, pH 5–10, 4–45 °C	y	y	y	y	y	y	y		PHB [40]
*Haloferax mediterranei* DSM 1411	NaCl 20%–25% (w/v) 37 °C	y			y	y	y		glycerol, starch	PHBV (18.21% ± 1.88% wt/wt) [10]

**Figure 3:**
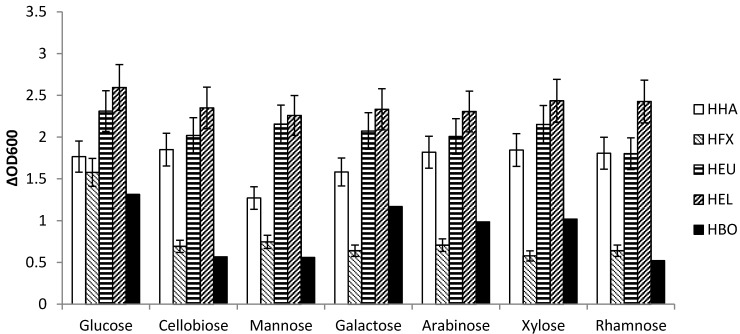
Screening for utilization of the seven most abundant sugars in SSL using the difference of OD_600_ as response. *H. eurihalina* (HEU) and *H. elongata* (HEL) are capable of utilizing all these sugars equally, whereas *H. boliviensis* (HBO) shows reduced growth on the most abundant sugar mannose, but also on cellobiose and rhamnose. *H. halophila* (HHA) is also less active on mannose compared to the other sugars, though the activity does not differ significantly. *Hfx. mediterranei* (HFX) shows less growth after 44 h, compared to the *Halomonas* species.

As suggested by the literature study *H. eurihalina* and *H. elongate* are capable of utilizing all investigated sugars equally (Figure 3), while *H. boliviensis* shows reduced growth on cellobiose, mannose and rhamnose, but grew on galactose. Surprisingly, *Hfx. mediterranei* and, to a certain degree, also *H. halophila* show reduced growth on mannose. With mannose being the most abundant sugar in the supplied SSL this finding is crucial.

**Figure 4:**
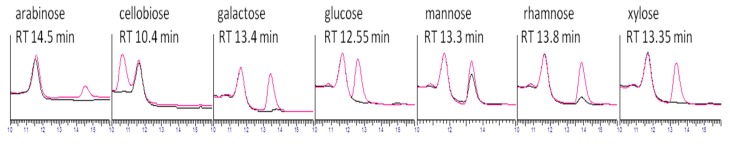
HPLC analysis was performed for *H. halophila* growing on the seven respective single sugars, arabinose, cellobiose, galactose, glucose, mannose, rhamnose and xylose. Samples were analyzed for their sugar content at inoculation (red line) and after 44 h of growth (black line) showing that all investigated sugars were utilized, except mannose was left after 44 h and also some rhamnose remained after 44 h. The peak at 11.7 min is a medium peak related to the high salt load.

Sugar measurements using HPLC analysis at time point zero and after 44 h also confirm these results. Example HPLC chromatograms are given for *H. halophil*a (Figure 4).

For *H. halophila*, it could be shown, that after 44 h of growth all sugars were utilized, but about 80% of the mannose remained after 44 h, indicating that, although it can be utilized, the specific uptake rate is lower. The same experiment was also conducted for the other strains. A summary of utilized sugars can be seen in Table 3.

**Table 3:** HPLC analysis was performed for all *Halomonas* strains growing on the seven respective single sugars, arabinose, cellobiose, galactose, glucose, mannose, rhamnose and xylose (at a concentration of 5 g·L^−1^). Samples were analyzed for their sugar content at inoculation and after 44 h of growth. A Y indicates the sugar was taken up, P indicates the sugar was partly taken up and N indicates the sugar was not utilized.

Strain	Arabinose	Cellobiose	Galactose	Glucose	Mannose	Rhamnose	Xylose
***H. boliviensis***	N	N	Y	Y	Y	N	Y
***H. elongata***	P	Y	Y	Y	Y	Y	Y
***H. eurihalina***	Y	Y	Y	Y	Y	Y	Y
***H. halophila***	Y	Y	Y	Y	P	Y	Y

Investigating the growth rate (μ) the individual strains reached on the various substrates, it could be shown, that μ of most strains is independent of the substrate. Only *H. boliviensis* shows slight differences in μ when grown on its favorite sugar glucose compared to growth on mannose (Table 4).

**Table 4:** Choice of substrate sugar has almost no effect on the maximal growth rate, μ_max_ (experimental error 10%).

Strain	μ_max_ [h^−1^] Arabinose	μ_max_ [h^−1^] Cellobiose	μ_max_ [h^−1^] Galactose	μ_max_ [h^−1^] Glucose	μ_max_ [h^−1^] Mannose	μ_max_ [h^−1^] Xylose
***H. boliviensis***	0.27	0.25	0.29	0.29	0.24	0.26
***H. elongata***	0.27	0.26	0.27	0.26	0.27	0.27
***H. eurihalina***	0.27	0.27	0.27	0.27	0.27	0.27
***H. halophila***	0.28	0.3	0.3	0.29	0.28	0.29
***H. mediterranei***	0.17	0.16	0.17	0.17	0.17	0.16

The microorganisms growing fastest on mannose, which is highly abundant in the investigated SSL, were *H. elongata*, *H. eurihalina* and *H. halophila*. As shown in Figure 3, the total OD_600_ values reached after 44 h of growth were higher in *H. halophila*, *H. elongata* and *H. eurihalina* than in the other two strains. Whether this faster growth also indicates a faster uptake of sugars had to be confirmed. This was confirmed by HPLC analysis (see Table 3 and Section 3.2.2).

#### 3.2.2. Effect of Mixed Substrate

As described in the above section, the sugar composition of lignocellulosic biomass is diverse. Therefore, on the one hand, how the species react to a mixed substrate, and on the other hand, what amount of sugar can be taken up before encountering media limiting conditions or a pH change that stops growth had to be investigated.

At first, the ability of the selected halophilic microorganisms to utilize high concentrations of sugar without pH control was tested. Respective media were supplemented with a sugar mixture at total sugar concentrations of 1 g·L^−1^, 3.6 g·L^−1^ and 36 g·L^−1^. The used sugar mixture, mimicking a diluted SSL (20% [w/w] dry matter), contained arabinose (0.9 g·L^−1^), cellobiose (0.24 g·L^−1^), galactose (2.6 g·L^−1^), glucose (6.1 g·L^−1^), mannose (22.5 g·L^−1^), rhamnose (0.19 g·L^−1^) and xylose (7.5 g·L^−1^). A second lag time occurred when only mannose was left and the culture needed time to adapt to mannose metabolism before growth could be resumed.

**Figure 5:**
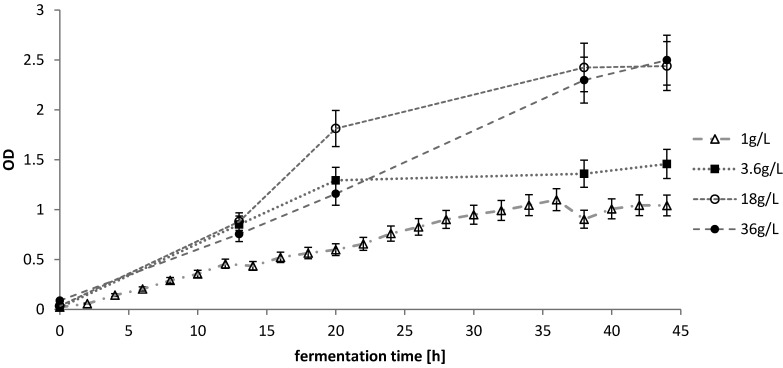
Effect of sugar concentration on selected *Halomona*s strain *H. halophila*.

Figure 5 shows the OD values from cultivations with *H. halophila*.

Although the growth characteristics for 18 g·L^−1^ and 36 g·L^−1^ show similar growth characteristics, it has to be considered that OD measurement is not directly linked to sugar uptake. On the one hand, oxygen limitation in shake flasks will allow only a certain number of cells to accumulate, which could then still utilize the remaining sugars to build storage substances like PHB. On the other hand, the complex medium could also support growth without using the sugar or after the sugar has been depleted.

**Figure 6:**
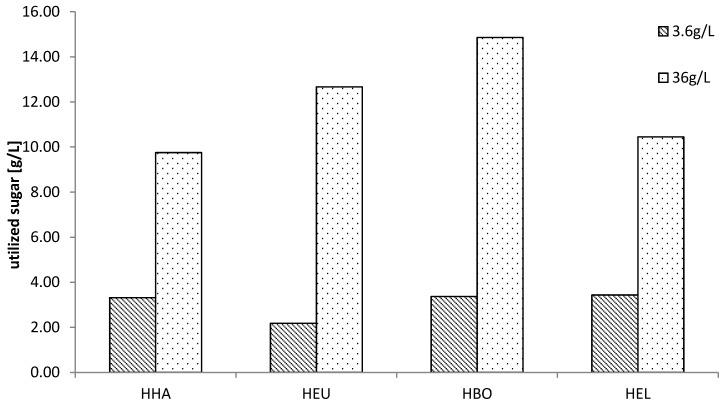
HPLC analysis was performed for *H. halophila, H. eurihalina, H. boliviensis* and *H elongata* growing on either 3.6 g·L^−1^ or 36 g·L^−1^ sugar mix, comprised of 2.5% arabinose, 0.5% cellobiose, 7% galactose, 16.5% glucose, 61% mannose, 0.5% rhamnose and 20% xylose. The bars show the utilized sugar (sum of all sugars) after 44 h.

The sugar uptake for all strains was tested for 3.6 g·L^−1^ and 36 g·L^−1^ after 44 h to investigate which strain was utilizing the highest amount of sugar in this time. The results can be seen in Figure 6.

HPLC analysis showed that there was little difference in sugar uptake by different strains at the lower concentration. At the high concentration, however, *H. halophila* and *H. elongata* were able to utilize around 10 g·L^−1^ sugar in 44 h. *H. eurihalina* was able to utilize more than 12 g·L^−1^ and *H. boliviensis* had the highest sugar uptake, with more than 14 g·L^−1^.

It was surprising that, although the OD values for *H. boliviensis* suggest low growth, it was able to utilize the highest amount of sugar within 44 h, compared to the other strains. This is a result of PHB production. As the target product, PHB is intracellular and is partly coupled to cell growth, however it is used for energy storage and, therefore, the amount of PHB inside the cell can vary. *H. boliviensis* is able to accumulate very high amounts of PHB, which explains the high sugar uptake.

These results indicate that *H. boliviensis* and *H. eurihalina* are promising candidates for industrial application, if they are able to grow on SSL.

**Figure 7:**
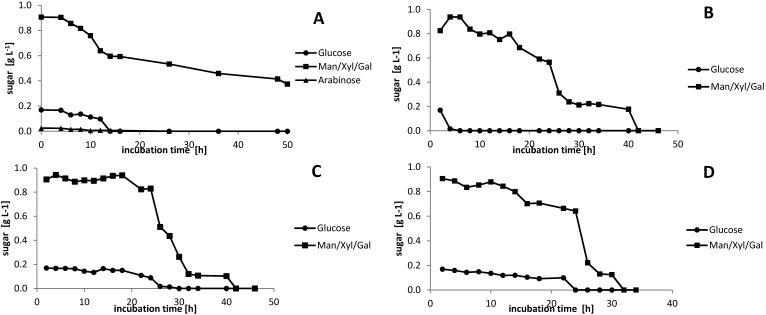
(**A**) *H. halophile*; (**B**) *H. boliviensis*; (**C**) *H. elongate*; and (**D**) *H. eurihalina* grown on 1 g·L^−1^ sugar mix, showing fermentation time *versus* sugar. The sugar mix is comprised of 25 mg arabinose, 7 mg cellobiose, 72 mg galactose, 169 mg glucose, 625 mg mannose, 5 mg rhamnose and 208 mg xylose. Due to the small amount of cellobiose, it could not be detected by the HPLC method used. Furthermore, arabinose could only be detected in the *H. halophila* medium, whereas it was not clearly detectable in the other media.

The sugar uptake was also investigated in detail for all strains. All the sugars (glucose, mannose, arabinose and xylose) were concurrently utilized during the fermentation, although the rate of sugar utilization was sugar specific (Figure 7).

With our HPLC method, it was not possible to separate mannose from xylose, rhamnose and galactose. However, it can be concluded from the single sugar results, that xylose, rhamnose and galactose were utilized in parallel to glucose. Figure 7A shows that 300 mg of the peak containing mannose, galactose, xylose and rhamnose (which roughly equals the sum of galactose, xylose and rhamnose) were utilized much faster than the remaining 600 mg, likely comprised of mannose. The results presented in Figure 7 suggest that *H. halophila* can utilize lignocellulosic sugars simultaneously. This simultaneous uptake and metabolism is a very desirable feature, since the sugar composition of lignocellulosic biomass is diverse.

### 3.3. Growth Tolerance against Diluted SSL

All strains were tested for their resistance to dilutions of the industrial 33.3% (w/w) dry matter (DM) SSL, containing approximately 5.4% (w/w) sugar. The respective media were supplemented with 3.33%. 6.66% and 16.65% (w/w) dry matter of SSL. The growth of cultures was monitored using OD, microscopy and HPLC analysis. All growing cultures were stained using the Nile Blue staining method to investigate the production of PHB.

**Figure 8:**
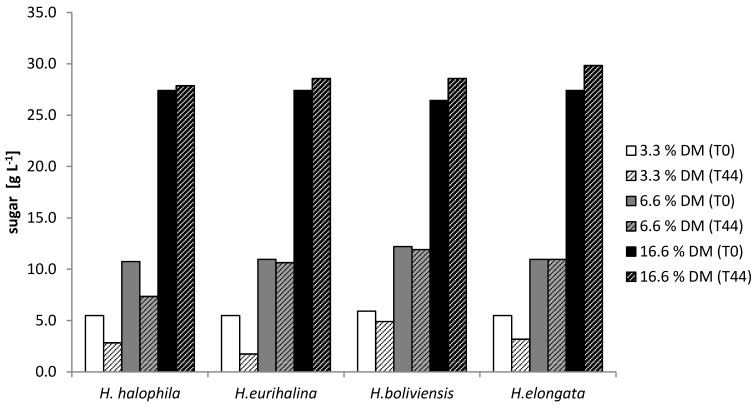
Utilization of sugars in diluted SSL after 44 h of cultivation. SSL was diluted in dH_2_O and supplemented with media components from a 10× stock solution. The starting pH value for all these fermentations was 7. *Hfx. mediterranei* could not be investigated in the HPLC, as no clear supernatant could be generated by centrifugation.

The HPLC results clearly show that, even diluted, SSL was not used readily (Figure 8).

Diluted SSL could not be utilized by *H. boliviensis*. *H. eurihalina* and *H. elongata* were able to thrive on SSL at a concentration of 3.3% (w/w) DM. Only *H. halophila* was able to utilize sugar in a 6.6% dry matter SSL solution (Figure 8).

The growth measurement data are in line with the sugar utilization results. As suggested by the HPLC results, there was no growth observed for *H. boliviensis* at any of the tested SSL concentrations. *H. eurihalina* and *H. elongata* showed growth at 3.3% (w/w) DM, while growth was observed for *H. halophila* in a 6.6% dry matter SSL solution.

**Figure 9:**
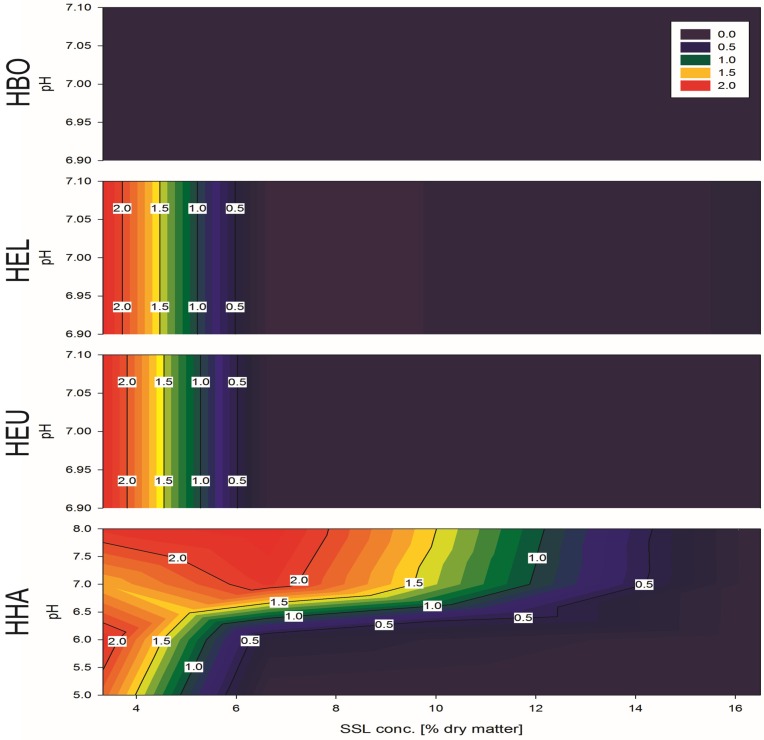
Growth on SSL as a sole monosaccharide source, color code indicating OD values. Investigating growth of *H. halophila* (HHA), *H. eurihalina* (HEU), *H. elongata* (HEL) and *H. boliviensis* (HBO) in respective media supplemented with 3.3%–16.65% (w/w) dry weight SSL at various pH values, resulting in growth on 3.3%, pH 7 for HEU and HEL; growth on 3.3%, pH 5, 6, 7 and 8 for HHA; and growth on 6.6%, pH 7 and pH 8 for HHA.

For better visualization, the obtained data were used to build a contour plot, giving estimation for the possible growth range of each strain (Figure 9).

HEU and HEL were able to grow on 3.3%, pH 7, whereas HHA was able to grow on 3.3% pH 5, 6, 7 and 8 and on 6.6% pH 7 and pH 8 (Figure 9). Although HFX was also investigated, the results were omitted from the above figure, as a rising OD value for HFX, which occurred at 6.6% and pH 6, was likely an artifact generated by precipitation. No viable cells could be found in the microscope, nor did cells, which were transferred back to their home medium, resume growth.

### 3.4. Effect of Known Inhibiting Substances in SSL on Growth

**Figure 10:**
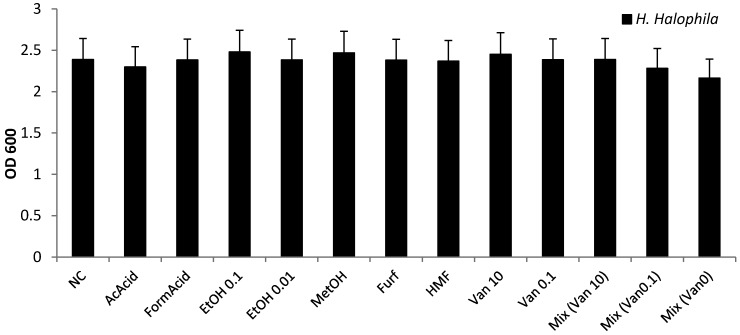
Effect of common inhibitors on growth of *H. halophila*. Concentrations roughly found in 30% (w/w) dry matter SSL (acetic acid 5.33 g·L^−1^, formic acid 157 mg L^-1^, ethanol 0.1% and 0.01%, methanol 150 mg·L^−1^, furfural 3 mg·L^−1^, HMF 39 mg·L^−1^, vanillin 0.1 mg·L^−1^ and 10 mg·L^−1^ as well as a Mix comprised of all inhibitors concentrated as in individual samples, EtOH 0.1%) were added to *H. halophila* medium supplemented with 18 g·L^−1^ of SSL sugar mix. Single inhibitors had only small effects on growth. None of these substances seems to explain the low tolerance of *H. halophila* to SSL.

In order to investigate why the tolerance against SSL was so low, selected common inhibitors from wood hydrolysate were tested for their effect on *H. halophila*. For these experiments, the inhibitor concentrations were chosen according to their concentration in 30% (w/w) dry matter SSL. The medium was supplemented with 18 g·L^−1^ sugar mix as available in the 16.5% (w/w) dry matter SSL used as highest SSL concentration. The inhibitor concentrations were adjusted to the amounts present in 30% (w/w) dry matter SSL, being methanol 150 mg·L^−1^, ethanol 0.1%, formic acid 157 mg·L^−1^, acetic acid 5.33 g·L^−1^, furfural 3 mg·L^−1^, and HMF 39 mg·L^−1^. None of the tested compounds showed an inhibitory effect. Vanillin was tested at a concentration of 10 mg·L^−1^as a model compound for phenolic inhibitors, and lignosulfonic acid was tested at 5 g·L^−1^, 10 g·L^−1^ and 25 g·L^−1^ (Figure 10).

Investigation of the generated data showed that at the used concentration, almost none of the investigated substances showed a significant inhibitory effect. Although slight differences can be seen, they are all within the 10.57% error margin that was determined as maximum observed standard deviation and was therefore applied as worst case to all bars.

These results were surprising, as an inhibitory effect has been reported for all of the tested substances. Organic acids, when not dissociated, have been reported to pass through the cell membrane inside the cell, where they can dissociate due to the higher intracellular pH. The intracellular pH thereby decreases, leading to acidification of the cytoplasm and thereby causing cell death [25,41].

The trend, that a concentration of 0.01% (w/w) ethanol did not show any effect, whereas 0.1% (w/w) ethanol facilitated growth can be explained by the assumption that ethanol can be used as a substrate.

Methanol has previously been shown to promote production of PHB in another PHB-producing strain [42]. Surprisingly, the same trend can be seen for vanillin, which *H. halophila* is able to utilize [18]. This is especially interesting in samples exposed to a mix of all substances, where the higher amount of vanillin is able to compensate for a seemingly synergetic effect of other inhibiting substances, as the mix of all inhibitors with no vanillin shows the least growth.

It has been reported that phenols harm the cell membrane [43]. However, the toxicity of phenols correlates with hydrophobicity [19,44] and vanillin was probably not the perfect model for an inhibitory phenolic compound. Therefore, an inhibitor study investigating a broad spectrum of phenols and lignin degradation products is being performed.

However, when interpreting these results, one has to keep in mind, that although testing high concentrations of individual phenols did not lead to inhibition in several studies, their removal from SSL facilitated growth. This could be explained by their solubility, which might be different in model medium compared to SSL, so that the microbes in the model substrates actually were exposed to lower concentrations than intended [21]. It is also not surprising that no toxic effect could be seen with the acids, as it has been shown before, that in a pH controlled system they did not inhibit growth of *Zymomonas* [45]. For several inhibiting substances, synergistic effects have been proposed [19], indicating that this truncated inhibitor mix, although containing example substances, just did not contain the right mix to be inhibitory.

For yeasts, the inhibitory effect of various salts has been investigated, showing that the inhibition of *S. cerevisiae* by different salts decreased in the following order: CaCl_2_, (NH_4_)_2_SO_4_ > NaCl, NH_4_Cl > KH_2_PO_4_ > MgCl_2_ > MgSO_4_ > KCl [46]. Comparing the toxicity of the anions sulfate and acetate in cultures of *Zymomonas mobilis*, showed that the acetate ions were more inhibitory than the sulfate ions [45]. Furthermore, Na^+^ and Cl^−^ inhibit growth of *Z. mobilis*, glucose consumption, and ethanol production, but the Na^+^ ion displayed more severe inhibitory effect [47]. The total amount of inorganic salts in the used SSL could be estimated; indicating that even after addition of up to 16.6% (w/w) DM SSL, the salt content was still well within the limits for the investigated strains. However, neither a detailed analysis of the salt fraction in the investigated SSL, nor a study on the effect of different salts on the used strains was available. Therefore it is unlikely, but possible, that the inorganic fraction has an inhibitory effect on the microorganisms.

### 3.5. Production of PHB on SSL

**Figure 11:**
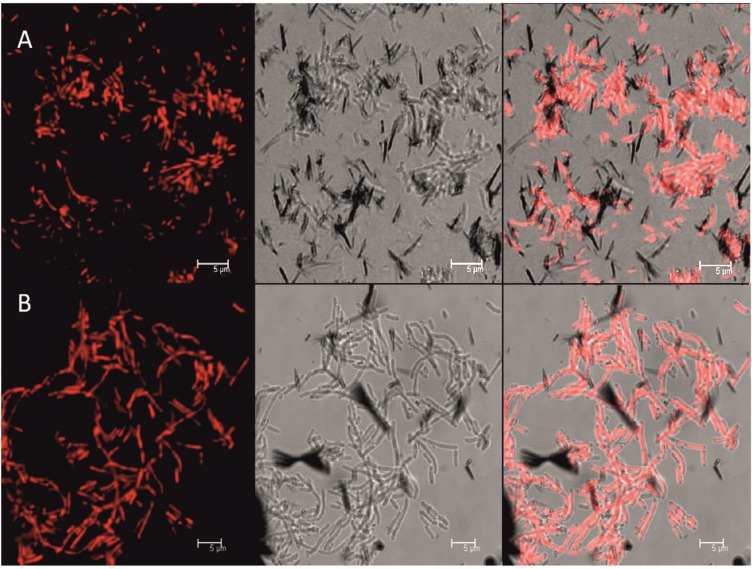
Fluorescence stain of H. halophila (**A**) grown on sugar mix and (**B**) grown on SSL (6.6% w/w dry matter), showing from left to right, the fluorescence image, the light microscopy image and an overlay of these two.

The production of PHB was investigated using fluorescence microscopy. Figure 11 shows the accumulation of PHB in *H. halophila* on 6.6% [w/w] dry matter SSL, which is similar to the production in home medium (Figure 11A,B).

For the other investigated species the PHB production on 3.3% (w/w) dry matter SSL was similar to the production on home medium, only *H. boliviensis* could not be cultivated on SSL at all.

## 4. Conclusions

The goal of the study was to show that the general concept of cultivating halophilic microorganisms for production of PHB on SSL is feasible. It could be demonstrated that, while *H. boliviensis* could not be cultivated on diluted spent sulfite liquor, *H. elongata*, *H. eurihalina* and *Hfx. mediterranei* could be cultivated on 3.3% w/w dry matter SSL, whereas *H. halophila* was able grow on 6.6% w/w dry matter SSL.

In order to investigate the production of biopolymers, only qualitative experiments were performed, showing that PHB was produced, regardless whether the culture was grown on model sugar or on SSL. This is already a very positive result and, to the authors’ knowledge, no comparable experiments have been reported in literature before.

These preliminary experiments left various open questions for further investigation:

- As the inhibitor experiments cannot explain the low tolerance of the halophilic microorganisms against SSL, the inhibitory effect of SSL must be caused by other, not yet investigated, factors like phenols other than vanillin or, less likely, the inorganic fraction.
- Detailed analysis of the phenolic and the inorganic fraction of pH adjusted SSL and the effect of single components on the investigated strains could be used for media adjustments that allow a much higher tolerance of the investigated halophilic strains to SSL.
- Can the *Halophiles* tolerate higher concentrations of SSL if they are slowly adapted to the inhibitors of this complex residual feedstock?
